# Influence of metformin on HIF-1 pathway in multiple myeloma

**DOI:** 10.1007/s43440-020-00142-x

**Published:** 2020-07-27

**Authors:** Kinga A. Kocemba-Pilarczyk, Sonia Trojan, Barbara Ostrowska, Małgorzata Lasota, Paulina Dudzik, Dorota Kusior, Marta Kot

**Affiliations:** 1 Medical Biochemistry, Jagiellonian University-Medical College, ul. Kopernika 7, 31-034 Kraków, Poland; 2 Department of Transplantation, Jagiellonian University Medical College, Kraków, Poland

**Keywords:** Metformin, Multiple myeloma, HIF-1 pathway

## Abstract

**Background:**

Multiple myeloma (MM) is defined as plasma cells malignancy, developing in the bone marrow. At the beginning of the disease, the malignant plasma cells are dependent on bone marrow microenvironment, providing growth and survival factors. Importantly, the recent studies pointed hypoxia as an important factor promoting progression of MM. In particular, hypoxia-triggered HIF-1 signaling was shown to promote chemoresistance, angiogenesis, invasiveness and induction of immature phenotype, suggesting that strategies targeting HIF-1 may contribute to improvement of anti-myeloma therapies.

**Methods:**

The Western Blot and RT-PCR techniques were applied to analyze the influence of metformin on HIF-1 pathway in MM cells. To evaluate the effect of metformin on the growth of MM cell lines in normoxic and hypoxic conditions the MTT assay was used. The apoptosis induction in metformin treated hypoxic and normoxic cells was verified by Annexin V/PI staining followed by FACS analysis.

**Results:**

Our results showed, for the first time, that metformin inhibits HIF-1 signaling in MM cells. Moreover, we demonstrated the effect of metformin to be mainly oxygen dependent, since the HIF-1 pathway was not significantly affected by metformin in anoxic conditions as well as after application of hypoxic mimicking compound, CoCl_2_. Our data also revealed that metformin triggers the growth arrest without inducing apoptosis in either normoxic or hypoxic conditions.

**Conclusions:**

Taken together, our study indicates metformin as a promising candidate for developing new treatment strategies exploiting HIF-1 signaling inhibition to enhance the overall anti-MM effect of currently used therapies, that may considerably benefit MM patients.

## Introduction

Multiple myeloma (MM), is one of the most common hematological malignancies described as a clonal expansion of plasma cells in the bone marrow associated with pancytopenia, renal failure and osteolytic bone disease. During the progression of the disease the MM cells are fully dependent on the bone marrow environment which comprises growth and survival factors for developing tumors. Interestingly, recent studies revealed that insufficient oxygenation in MM infiltrated bone marrow constitutes an important factor enhancing the aggressiveness of this malignancy [1]. In particular, hypoxia in MM was reported to enhance tumor initiation, upregulate genes expressed at the immature B-cell stage (induction of immature phenotype) [2, 3] and confer the resistance to proteasome inhibitors [2]. Moreover, oxygen deficiency was described as a factor stimulating migration and homing of malignant plasma cells to the new BM niches [4], promoting osteolytic bone destruction and significantly contributing to MM-induced angiogenesis [5–7]. In consequence, several studies have reported the inhibition of HIF-1, critical regulator of cellular responses to low oxygen levels, as the strategy to target hypoxia-induced alterations in malignant plasma cells [7–9]. Nevertheless, despite promising rationale, none of HIF-1 inhibitors has so far been approved for treating cancer patients, due to the limited therapeutic efficacy as well as toxic side effects [10]. Interestingly, however, the recent studies revealed that metformin, commonly used anti-diabetic drug, inhibits the HIF-1 stabilization/HIF-1 signaling in human hepatocellular carcinoma [11], cervical squamous carcinoma cells [12] and oral squamous cell carcinoma [13]. On the other hand, it has been shown that metformin treatment, longer than 2 years, exerts protective effect against the development of MM in diabetic patients diagnosed with MGUS [14]. Taking all the above into consideration, we decided to verify whether metformin influences the HIF-1 pathway in malignant plasma cells.

## Materials and methods

Metformin (1,1-Dimethylbiguanide, Hydrochloride) was purchased from Santa Cruz Biotechnology.

Cobalt Chloride (CoCl_2_), Taxol, Ponceau S, nitrocellulose membrane and MTT (Methyl-Thiazolyl-Tertazolium) were purchased from Sigma-Aldrich. Hypoxyprobe (1000 mg pimonidazole HCl plus 2 × 1.0 ml of 4.3.11.1 mouse Mab) was purchased from Hypoxyprobe Inc. (121 Middlesex Turnpike Burlington MA 01803 USA).

In each experiment the fresh metformin solution (100 mM) was prepared by reconstituting metformin into serum–free RPMI medium.

### Cell culture

Multiple Myeloma cell lines, L363 and RPMI 8226, were cultured in RPMI medium 1640 (Invitrogen Life Technologies, Carlsbad, CA) supplemented with 10% clone I serum (EURx, Poland, PL), 100 units/ml penicillin (Sigma-Aldrich,), and 100 µg/ml streptomycin (Sigma-Aldrich). Cells were cultured in normoxic, hypoxic and anoxic conditions. Initially cells were plated in 6 wells plate dedicated for suspension cells (Greiner Bio-One International) in the density 5 × 10^5^ cells cells/ml in 2 ml of culture medium. The cells were pre-incubated with metformin for 1 h and subsequently cultured in hypoxic or anoxic conditions for 24 h. To reach oxygen-deprived environment the inflatable hypoxia chamber (Modular Incubator Chamber, Billups-Rothenberg, Inc.) and the proper gas mixtures were used. To obtain hypoxic conditions the chamber was flushed for 20 min with gas mixtures containing 5% CO_2_, 1% O_2_ and 94% N_2_ at the flow rate of 25 liters per minute. For reaching the anoxic conditions the gas mixture composed of 5% CO_2_ and 95% N_2_ was flushed through the chamber for 10 min and next the chamber was placed in conventional incubator to allow the culture medium to de-gas. After 1 h the flush with anoxic gas was repeated for another 10 min and the cells were placed back to the incubator for next 24 h. In accordance with literature and the manufacturer instructions (Billups-Rothenberg), 10 min flushing at the flow rate 25 liters per minute is sufficient to eliminate most if not all oxygen in the chamber, allowing thereby to achieve the anoxic conditions defined in literature as severe hypoxia, with the estimated residual oxygen level below (< 0.01% O_2_). For Cobalt Chloride (CoCl_2_) experiment the cells were seeded in 6 well plate dedicated for suspension cells (Greiner Bio-One International) in the density 5 × 10^5^ cells/ml in 2 ml of culture medium. The cells were pre-incubated with metformin (5 mM) for 1 h and subsequently the CoCl_2_ in the final concentration of 100 µM was applied for additional 24 h. To access the oxygenation status in short-term hypoxia the cells were seeded in 6 well plate dedicated for suspension cells (Greiner Bio-One International) in the density 5 × 10^5^cells cells/ml in 2 ml of culture medium and upon 1 h pre-incubation with metformin (5 mM) the hypoxyprobe (Hypoxyprobe, Inc. 121 Middlesex Turnpike Burlington, MA 01803 USA) was added at the final concentration of 100 µM following the 5 h incubation in normoxic and hypoxic conditions.

### RT-PCR

RNA was isolated from L363 and RPMI 8226 cells using GenElute™ Mammalian Total RNA Miniprep Kit (Sigma-Aldrich) according to the manufacturer’s instructions. Subsequently, the quality and the quantity of isolated ribonucleic acid was measured using a NanoDrop ND-1000 Spectrophotometer (NanoDrop Technologies, Wilmington, DE). The synthesis of cDNA form 1000 ng of isolated RNA was performed using NG dART RT kit (EURx, Poland, PL). For PCR reaction 1.5 ul of cDNA was added to the mixture composed of 10 µl of Color OptiTaq PCR Master Mix (2x) (EURx, Poland, PL), 10 µl of dH_2_O (EURx, Poland, PL) and 0.2 µl of each 10 µM primers (Sigma-Aldrich). The following primers were used: BNIP3 (BCL2 Interacting Protein 3) forward (5′-CACCTCGCTCGCAGACACCAC-3′), BNIP3 reverse (5′-GAGAGCAGCAGAGATGGAAGGAAAAC-3′), PFKFB4 (6-Phosphofructo-2-Kinase/Fructose-2,6-Biphosphatase 4) forward (5′-GGGATGGCGTCCCCACGGG-3′), PFKFB4 reverse (5′-CGCTCTCCGTTCTCGGGTG-3′), CAIX (Carbonic Anhydrase IX) forward (5′-TACAGCTGAACTTCCGAGCG-3′), CAIX reverse (5′-CTAGGCTCCAGTCTCGGCTA-3′), ANGPTL4 (Angiopoietin-like 4) forward (5′-AGATGAATGTCCTGGCGCAC-3′), ANGPTL4 reverse (5′-GGATCCTGCTGTTCTGAGCC-3′), HPRT1 (Hypoxanthine phosphoribosyltransferase-1) forward (5′-TGGCGTCGTGATTAGTGATG-3′), HPRT1 reverse (5′-TATCCAACACTTCGTGGGGT-3′).

The PCR was performed in MJ Research PTC-200 Thermal Cycler. For all primers used in the study the same PCR conditions were applied: initial denaturation at 95 ℃ for 5 min; 95 ℃, 58 ℃, 72 ℃, each for 30 s for 30 cycles. To complete the reaction 10 min incubation at 72 ℃ was applied. Electrophoresis on the agarose gel (1.5% w/v), containing ethidium bromide, was used for PCR product visualization. Housekeeping gene, HPRT1, was used to normalize the results.

### Western blot

For the protein analysis L363 and RPMI 8226 cells were cultured in normoxic, hypoxic, anoxic conditions for 24 h and in normoxic conditions in the presence and absence of Cobalt Chloride (100 µM). Subsequently the cells were lysed directly in sample buffer and total protein concentration was measured by of Lowry assay with modification of Peterson [15]. Next, the 30 µg or 60 µg of protein lysate was separated by SDS/10% PAGE, and subsequently blotted. Nitrocellulose membranes (Sigma-Aldrich) were blocked 1 h at room temperature in low fat milk in TBST (Tris-buffered saline, 0.1% Tween20) and subsequently incubated overnight at 4 ℃ with primary anti-HIF-1 alpha antibody (HIF-1α) (#3716, Cell Signaling), anti-β-actin antibody (Clone AC-15, Sigma-Aldrich) and anti-pimonidazole (Hypoxyprobe kit #4.3.11.3) following by 1 h incubation in room temperature with appropriate secondary HRP-linked antibodies (#7076 and #7074, Cell Signaling). For visualization of protein expression the ECL™ Western Blotting Detection Reagents (GE, Healthcare) were used. Chemiluminescence signal was detected using Bio-Rad ChemiDoc™ XRS + System (Bio-Rad). The Ponceau S staining was used as a loading control for Western Blot detecting pimonidazole–protein adducts. Briefly, the nitrocellulose membrane, directly after blocking was stained around 1 min with Ponceau S dye 0.5% (w/v) (Sigma-Aldrich, ThermoFisher Scientific) in 1% (v/v) acetic acid (Sigma-Aldrich, ThermoFisher Scientific). The excess of the dye was removed by washing the membrane with distilled water and subsequently the membrane was photographed using Bio-Rad ChemiDoc™ XRS + System (Bio-Rad). Prior to the blocking procedure the Ponceau S stain was removed from the protein bands by washing the membrane several times with TBST solution.

### MTT (Methyl-Thiazolyl-Tertazolium) assay adjusted for cells cultured in suspension

MTT assay was used to analyze the response of malignant plasma cells to varying concentrations of metformin in normoxic and hypoxic conditions. Briefly, myeloma cells) were seeded into 96-well plates dedicated for suspension cells (Greiner Bio-One International) in final concentration 3 × 10^4^ cells in 200 µl of cell culture medium and subsequently cultured for 3 days with varying concentrations of metformin (25 mM, 12.5 mM, 6.25 mM, 3.125 mM, 1.56 mM). In each experiment the cells were initially pre-incubated with metformin for 1 h and subsequently cultured for 72 h in normoxic and/or hypoxic conditions. Next, 10 μl of the dye (Methyl-Thiazolyl-Tertazolium 5 mg/ml) (Sigma-Aldrich) was added to each well and the cells were left in the standard incubator (37 °C degrees) for 3 h. Subsequently, the cells were lysed in the lysis buffer (10% SDS in 0.01 M HCl) and left for additional 24 h in the standard incubator for the lysis. When the lysis was complete, the optical density at 570 nm was measured.

### Cell number assessment using Bürker chamber

The cell number assessment using Bürker chamber was used to analyze the response of L363 and RPMI 8226 cells to different concentration of metformin (10 mM, 5 mM, 2.5 mM) in normoxic and hypoxic conditions. Briefly, myeloma cells were seeded into 96-well plates dedicated for suspension cells (Greiner Bio-One International) in the final concentration 3 × 10^4^ cells in 200 µl of cell culture medium and subsequently cultured for 3 days with varying concentrations of metformin (10 mM, 5 mM, 2.5 mM). In each experiment the cells were initially pre-incubated with metformin for 1 h before culturing for 72 h in normoxic and/or hypoxic conditions. Upon indicated time point, 10 μl of cell suspension was mixed with 10 μl of 0.4% Trypan Blue (Gibco) and applied into the Bürker chamber. To determine the cell density per ml in each well, the number of living cells (trypan blue negative) in 3 corner squares were counted and the average was multiplied by 10.000 and the dilution factor.

### Flow cytometry analysis

For flow cytometry analyses MM cell lines L363 and RPMI 8226 were seeded in 6-well plates at a density of 3 × 10^5^ cells per well and subsequently cultured for 48 h in normoxic and hypoxic conditions, in the presence or absence of 5 mM metformin concentration. After indicated time point the cells were stained with Annexin V-FITC and Propidium Iodide (PI) (FITC Apoptosis Detection Kit, BD Pharmingen) and analyzed by FACS (Attune NxT, Life Technologies) as previously described [12]. To properly determine the level of compensation the single stained samples and positive control were used in each experiment. As positive control for the experimental setup the multiple myeloma cells treated 16 h with 100 nM of Taxol (the compound inducing apoptosis as well as necrosis), were used.

### Statistical analysis

As for western blot and RT-PCR results the comparisons were made between hypoxic cells treated with metformin and hypoxic cells not treated with metformin, anoxic cells treated with metformin and anoxic cells not treated with metformin, CoCl_2_ (100 µM) stimulated cells treated with metformin and CoCl_2_ (100 µM) stimulated cells not treated with metformin. In each experiment performed in oxygen diminished environment the control cells (normoxic ones) were used in parallel, as a confirmation of proper HIF-1 pathway induction. In experiment with CoCl_2_ (100 µM) the cells not treated with CoCl_2_ were used as a confirmation of proper HIF-1 pathway stimulation by CoCl_2_. Densitometry values of Western blot and RT-PCR bands intensity were normalized to β-actin and HPRT1, respectively, and each relative densitometry value was presented as the mean from three independent experiments. Student *t* test was used to evaluate significant differences between the level of stabilized HIF-1 alpha in the absence of metformin (in hypoxic condition, anoxic condition and in the presence of CoCl_2_ (100 µM)) and the level of stabilized HIF-1 alpha in the presence of metformin (in hypoxic condition, anoxic condition and in the presence of CoCl_2_ (100 µM) as well as expression of particular gene in the absence of metformin (in hypoxic condition, anoxic condition and in the presence of CoCl_2_ (100 µM) and expression of the same gene in the presence of metformin (in hypoxic condition, anoxic condition and in the presence of CoCl_2_ (100 µM). Statistical analysis was performed by GraphPad Prism 5.0 software (GraphPad Software Inc., San Diego, CA, USA). For analysis purpose the *p* values < 0.05 were considered statistically significant, whereas *p* values between 0.05 and 0.1 were considered as an indication of the trend. As for the growth assay (MTT and trypan blue) the response to metformin concentration range was compared between normoxic and hypoxic cells. To verify the significance of the influence of hypoxia and metformin concentration on the relative growth of MM cells, the multiway ANOVA was used (Statistica 12, StatSoft Poland). The multiway ANOVA with Fisher’s least significant difference (LSD) posthoc test was also used to test the difference in the response of normoxic and hypoxic MM cells to particular metformin concentration. In addition, the multiway ANOVA was used to test the difference in the in the number of living cells, early apoptotic cells and late apoptotic cells between normoxic and hypoxic MM cells cultured in the presence and absence of metformin.

## Results

### Influence of metformin on HIF-1 pathway activation induced by hypoxia, anoxia and Cobalt Chloride

**Fig. 1:**
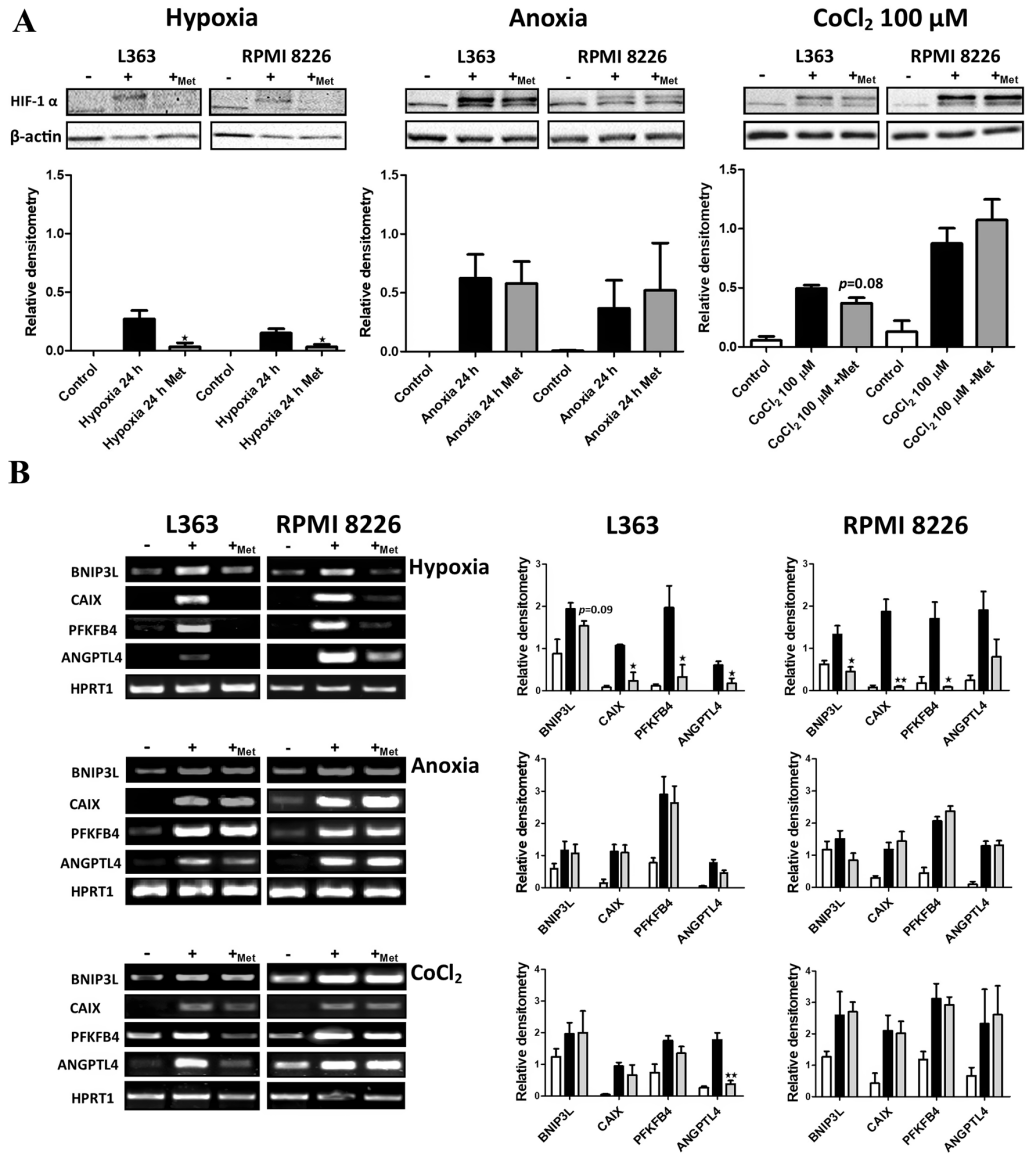
Influence of metformin on HIF-1 pathway activation induced by hypoxia, anoxia and cobalt chloride. **a** Influence of metformin on HIF-1 alpha stabilization. Upper panel: Multiple Myeloma cell lines were cultured for 24 h in different conditions in the presence and absence of metformin (5 mM) and subsequently HIF-1 alpha subunits accumulation was verified using the Western Blot. β-actin is shown as an internal control for equal loading. Lower panels: densitometry analysis of Western blot bands intensity normalized to β-actin. Each relative densitometry value is the average from three independent experiments. The mean ± SEM is shown. Student *t* test was used to evaluate influence of metformin on HIF-1 alpha stabilization. *p < 0.05 by student’s *t* test, *p* value between 0.05 and 0.1, by student’s *t* test was given as an indication of the trend. In all presented Western blot 30 µg of protein lysate was applied. **b** Influence of metformin on HIF-1 transcriptional response. Left panel: multiple myeloma cell lines, L363 and RPMI 8226, were cultured for 24 h in different conditions in the presence and absence of metformin (5 mM) and the influence of metformin on HIF-1 transcriptional response was assessed by RT-PCR. HPRT1 was used as an internal control for equal loading. Right panel: densitometry analysis of RT-PCR bands intensity normalized to HPRT1. Each relative densitometry value is the average from three independent experiments. The mean ± SEM is shown. Student *t* test was used to evaluate influence of metformin on HIF-1 triggered gene expression. *p < 0.05 by student’s *t* test, **p < 0.01 by student’s *t* test, *p* value between 0.05 and 0.1, by student’s *t* test, was given as an indication of the trend

To verify if metformin can block oxygen dependent HIF-1 alpha stabilization we initially analyzed the response of multiple myeloma cell lines, L363 and RPMI 8226, to hypoxic conditions in the presence and absence of 5 mM metformin. As shown in Fig. 1a (left panel), accumulation of HIF-1 alpha subunit was observed in L363 and RPMI 8226 cell lines cultured in hypoxic conditions without 5 mM of metformin, whereas in the presence of metformin (5 mM) HIF-1 alpha subunit was not stabilized. To evaluate influence of metformin on HIF-1 alpha stabilization the densitometry signal of HIF-1 alpha subunit in hypoxic cells cultured with and without metformin (5 mM) was compared using Student *t* test. In L363 cell line the level of stabilized HIF-1 alpha was significantly lower in hypoxic cell in the presence of metformin (5 mM) (*p* = 0.0413, *t*_4_ = 2.967) as well as in RPMI8226 cells (*p* = 0.0489, *t*_4_ = 2.798). To further determine if the reduction of HIF-1α subunit accumulation attenuates HIF-1 transcriptional activity, RT-PCR assay was performed. The genes selected for that analysis were proven previously as the targets of HIF-1 transcription factor and their induction, as a consequence of HIF-1 nuclear accumulation, has been reported in malignant plasma cells [5, 8]. In accordance with the protein data, in both tested cell lines, cultured in hypoxic conditions, the induction of target genes was observed, while in the presence of metformin (5 mM) the expression level of analyzed genes was comparable with the control cells (1B, upper panel). To evaluate the influence of metformin on the expression of HIF-1 target genes the densitometry signal in hypoxic cells cultured with and without metformin (5 mM) was compared using student *t* test. In L363 cell line the densitometry signal for *CAIX*, (*t*_4_ = 4.14, *p* = 0.0147), *PFKFB4* (*t*_4_ = 3.030, *p* = 0.0388) and *ANGPTL4 *(*t*_4_ = 2.833, *p* = 0.0472) genes was significantly lower in hypoxic cells treated with metformin in comparison to untreated hypoxic ones. As for *BNIP3* gene in L363 cells, the *p* value was below 0.1 (*t*_4_ = 2.144, *p* = 0.0987) what indicates the trend for the lower expression in hypoxic cells in the presence of metformin in comparison to expression level in hypoxic cells cultured without metformin. The similar data was obtained in RPMI8226 cells, where densitometry signal was significantly lower for *BNIP3*, (*t*_4_ = 3.685, *p* = 0.0211), *CAIX* (*t*_4_ = 6.116, *p* = 0.0036) and *PFKFB4* (*t*_4_ = 4.160, *p* = 0.0142) genes in cells cultured in hypoxic condition in the presence of metformin (5 mM) in comparison to cells cultured in hypoxic conditions without metformin.

To verify if oxygen plays indeed an important role in the mechanism responsible for prevention of nuclear HIF-1 accumulation by metformin in hypoxic multiple myeloma cells, we established the non-physiological anoxia, meaning almost complete oxygen-deprived culture environment and in this experimental setup we determined the influence of metformin on HIF-1 alpha stabilization. As shown in Fig. 1a (middle panel), by contrast to hypoxic conditions, in anoxia metformin was not able to attenuate the HIF-1 alpha accumulation and in consequence the HIF-1 transcriptional response, what indirectly suggests that complex I impairment and subsequent oxygen shift from mitochondria to cytosol may play a role in HIF-1 signaling inhibition in metformin treated hypoxic myeloma cells. To further prove that the effect of metformin on HIF-1 signaling is mainly oxygen dependent we performed the experiment with Cobalt Chloride, which inhibits prolyl hydroxylases leading to the accumulation of HIF-1α and in consequence triggers the transcriptional activity of HIF-1. Interestingly, in L363 cell line we observed the trend *p* < 0.1 for the lower level of stabilized HIF-1 alpha subunit (*t*_4_ = 2.318, *p* = 0.0814) in the presence of metformin (Fig. 1a, right panel) in comparison to cells treated with CoCl_2_ in the absence of metformin. As for transcriptional activity, only the CoCl_2_ triggered expression of *ANGPTL4* was significantly diminished (*t*_4_ = 5.637, *p* = 0.0049) by the presence of metformin. In the second cell line, RPMI 8226 we did not observe the influence of metformin on the response to Cobalt Chloride at the protein as well as at mRNA level (Fig. 1a right panel and 1b lower panel). Altogether, experiment presented in Fig. 1 strongly suggests that the effect of metformin on HIF-1 signaling in myeloma cells is mainly oxygen dependent.

### Kinetic analysis of metformin influence on HIF-1 alpha stabilization in hypoxic multiple myeloma cells.

**Fig. 2:**
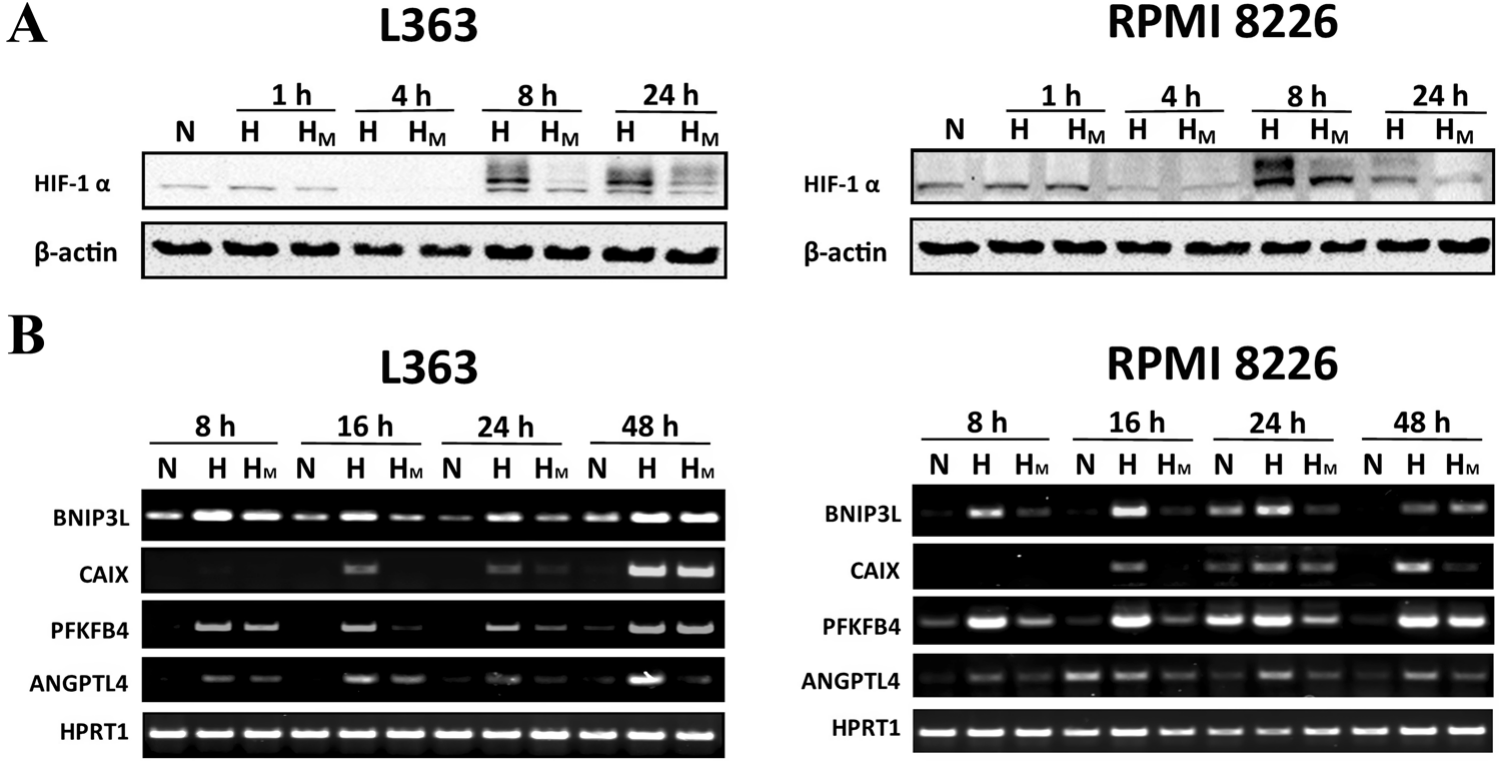
Kinetic analysis of metformin activity in hypoxic multiple myeloma cells. **a** Kinetic analysis of metformin influence on HIF-1 alpha stabilization in hypoxic multiple myeloma cells. L363 and RPMI 8226 cell lines were cultured in normoxic and hypoxic conditions for 1 h, 4 h, 8 h and 24 h in the presence and absence of metformin (5 mM). HIF-1 alpha subunits accumulation was verified using the Western Blot. β-actin is shown as an internal control. For this particular analysis 60 µg of protein lysate was used. The experiment was performed twice with the similar results. **b** Kinetic analysis of metformin influence on HIF-1 transcriptional response in hypoxic multiple myeloma cells. L363 and RPMI 8226 cell lines were cultured in normoxic and hypoxic conditions for 8 h, 16 h, 24 h and 48 h in the presence and absence of metformin (5 mM). The influence of metformin on transcriptional response was assessed by RT-PCR. HPRT1 was used as an internal control for equal loading. The experiment was performed twice with the similar results

To further determine if metformin continuously inhibits hypoxic HIF-1 alpha stabilization and HIF-1 dependent signaling in the studied time frame, we perform kinetic analysis for HIF-1 pathway activation. As it has been shown in Fig. 2, we did not observe the HIF-1 alpha stabilization upon 1 h and 4 h of culture in hypoxia chamber, what can be explained by the presence of residual oxygen in culture medium, still sufficient to prevent the HIF-1 alpha stabilization. Of note, after 8 and 24 h of culture in hypoxic conditions, stabilization of HIF-1 alpha subunit was clearly visible in both studied cell lines, in parallel with a decrease in its level in the presence of metformin. Kinetic analysis of transcriptional response after 8 h,16 h, 24 h and 48 h of hypoxic culture revealed that the metformin effect is constant up to 24 h for analyzed transcripts, whereas after 48 h the metformin effect was visible only for *ANGPTL4* (L363 and RPMI 8226) and *CAIX* (RPMI 8226) gene (Fig. 2b).

### The influence of metformin on oxygenation status of hypoxic multiple myeloma cells

**Fig. 3:**
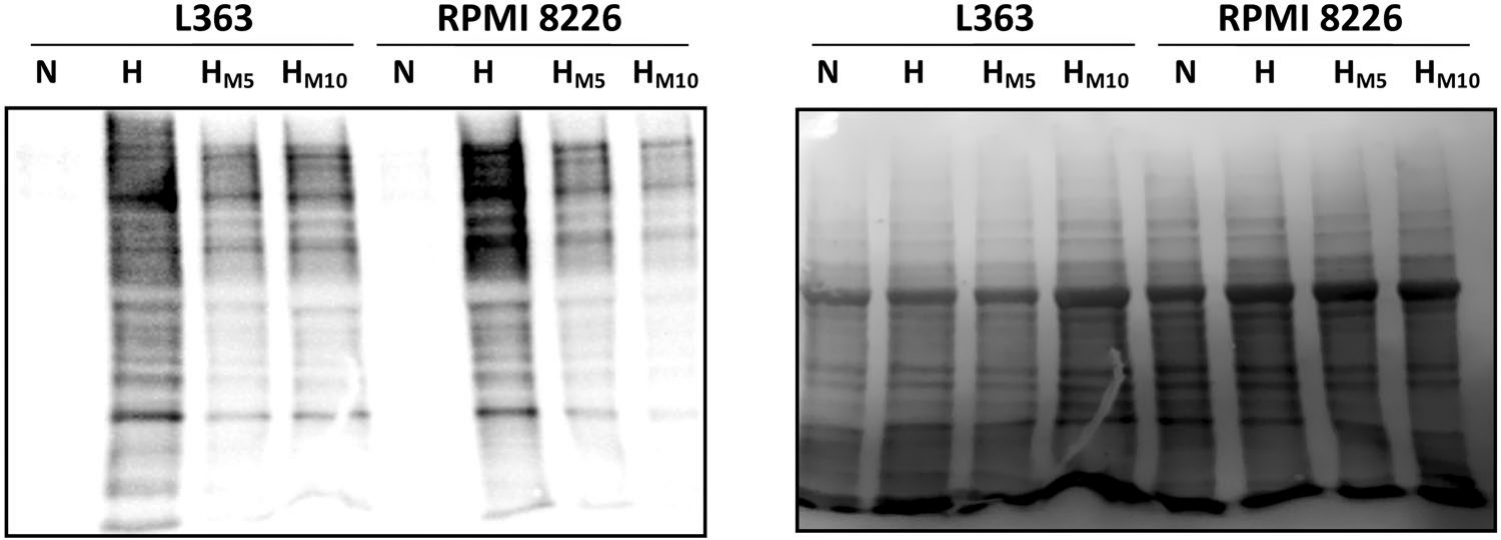
Influence of metformin on oxygenation status of hypoxic multiple myeloma cells. Left panel: MM cell lines, RPMI 8226 and L363, were cultured with 100 µM pimonidazole (hypoxyprobe) in normoxic (N) and hypoxic conditions (H) for 5 h in the presence and absence of 5 mM metformin (H_**M5**_) and 10 mM metformin (H_**M10**_). The formation of pimonidazole–protein adducts was detected using Western Blot. Right panel: the Ponceau S stained membrane is shown as an internal control for equal protein loading. The experiment was performed three times with the similar results

To further verify, if during short hypoxia intervals the metformin treated cells have indeed the higher oxygenation status in comparison to untreated ones, the oxygen tension within the cells was assessed using pimonidazole, the compound forming the adducts with intracellular proteins in hypoxic conditions, what can be further visualized by Western Blot. As shown in Fig. 3, the staining intensity for protein–pimonidazole adducts in hypoxic cells was lower in metformin treated cells in comparison to untreated ones in both analyzed cell lines, what altogether may indicate that metformin increases the oxygenation status of myeloma cells in oxygen diminished environment, most probably by attenuation of oxygen consumption.

### The influence of metformin concentration on the multiple myeloma growth in normoxic and hypoxic conditions

**Fig. 4:**
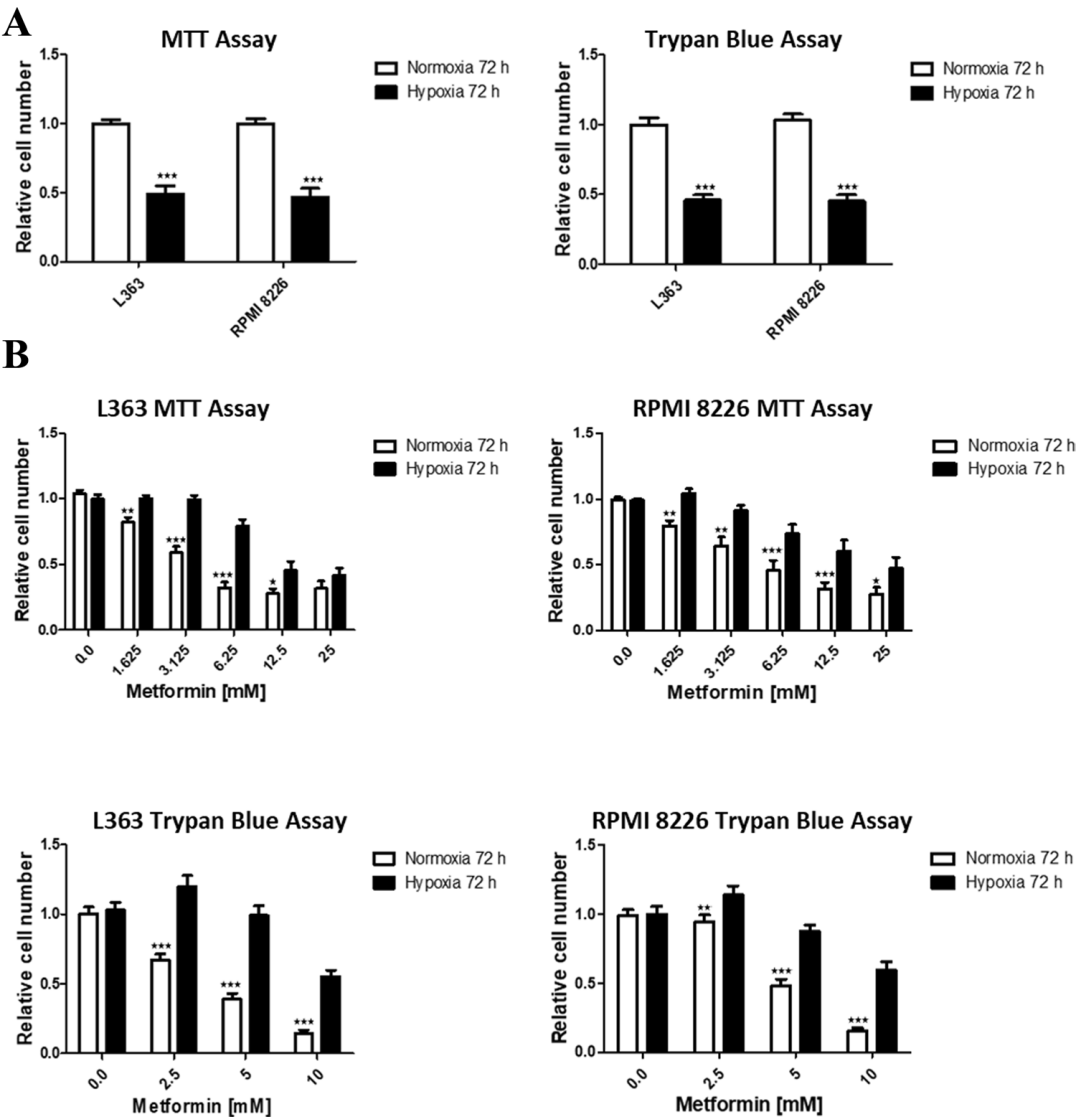
Influence of metformin on growth of multiple myeloma cells in hypoxic and normoxic conditions. **a** The influence of hypoxia on the growth of multiple myeloma cells. MM cell lines, RPMI 8226 and L363, were cultured in normoxic and hypoxic conditions for 72 h. Left panel: the absorbance of normoxic cells was normalized to 1 and subsequently the absorbance of hypoxic ones was normalized to this value. Right panel: the number of cell in normoxic conditions was normalized to 1 and subsequently the number of hypoxic ones was normalized to this value. The mean ± SEM of at least three independent experiments is shown. Student *t* test was used to evaluate the differences between the cell growth in normoxic and hypoxic conditions ***p < 0.001 by student’s *t* test. **b** The influence of the range of metformin concentration on the multiple myeloma growth in normoxic and hypoxic conditions. MM cell lines, RPMI 8226 and L363, were cultured in normoxic and hypoxic conditions with the range of metformin concentrations for 72 h. Upper panel: the absorbance of normoxic and hypoxic cells cultured without the presence of metformin was normalized to 1 and subsequently the absorbance of normoxic and hypoxic cells treated with metformin was normalized to its corresponding value. Lower panel: the number of normoxic and hypoxic cells cultured without the presence of metformin was normalized to 1 and subsequently the number of normoxic and hypoxic cells treated with metformin was normalized to its corresponding value. The mean ± SEM of at least three independent experiments is shown. Multiway ANOVA was used to evaluate the influence of hypoxia and metformin on L363 and RPMI 8226 cells. The significant differences in response to metformin between hypoxic and normoxic cells are indicated on the graph (by multiway ANOVA with Fisher’s least significant difference (LSD) posthoc test). *p < 0.05, **p < 0.01, ***p < 0.001

To determine the influence of metformin on the growth of malignant plasma cells, we treated L363 and RPMI 8226 MM cell lines by the range of metformin concentrations in hypoxic and normoxic conditions. Although the effect of metformin on the growth of malignant plasma cells in normoxic conditions has already been published [16], according to our knowledge such analysis has not yet been performed in hypoxia. For correct analysis of metformin effect on cellular growth, the two different assays have been used, MTT assay, measuring the metabolic activity and direct cell counting in Bürker chamber, using trypan blue exclusion assay. As it has been shown in Fig. 4a, in hypoxic conditions after 72 h of treatment, in both cell lines, using two different assays, we observed the decrease in the number of cells (upper panel). The significance of the difference between the cells number in hypoxic and normoxic conditions (L363 MTT assay *t*_22_ = 7.677 *p* < 0.0001, RPMI8226 MTT assay *t*_22_ = 7.276 *p* < 0.0001, L363 trypan blue assay *t*_40_ = 8.823 *p* < 0.0001, RPMI 8226 trypan blue assay *t*_40_ = 9. 295 *p* < 0.0001) was confirmed using student *t* test. Interestingly, as shown in Fig. 4b, according to MTT and trypan blue exclusion assay, the response of MM cells to metformin was dose-dependent, nevertheless the effect on cell growth was diminished in hypoxic environment in comparison to normoxia. To verify the significance of the influence of hypoxia and metformin concentration on MM cells, the multiway ANOVA was applied. According to the test results, the significant influence of hypoxia as well as metformin concentration on malignant plasma cells was confirmed for both tested cell lines using two different assays, MTT and trypan blue, (L363 MTT assay (*F*_11.144_ = 46.25, *p* < 0.001), L363 trypan blue assay (*F*_7.160_ = 46.61, *p* < 0.001), RPMI8226 MTT assay (*F*_11.144_ = 22.44, *p* < 0.001) and RPMI8226 trypan blue assay (*F*_7.160_ = 43.57, *p* < 0.001). Moreover, the multiway ANOVA with Fisher’s Least Significant Difference (LSD) posthoc test confirmed the significant difference in the response to particular metformin concentrations between normoxic and hypoxic myeloma cells using MTT assay as well as the trypan blue assay (Fig. 4b).

### Effect of metformin on apoptosis induction in malignant plasma cells in normoxic and hypoxic conditions

**Fig. 5:**
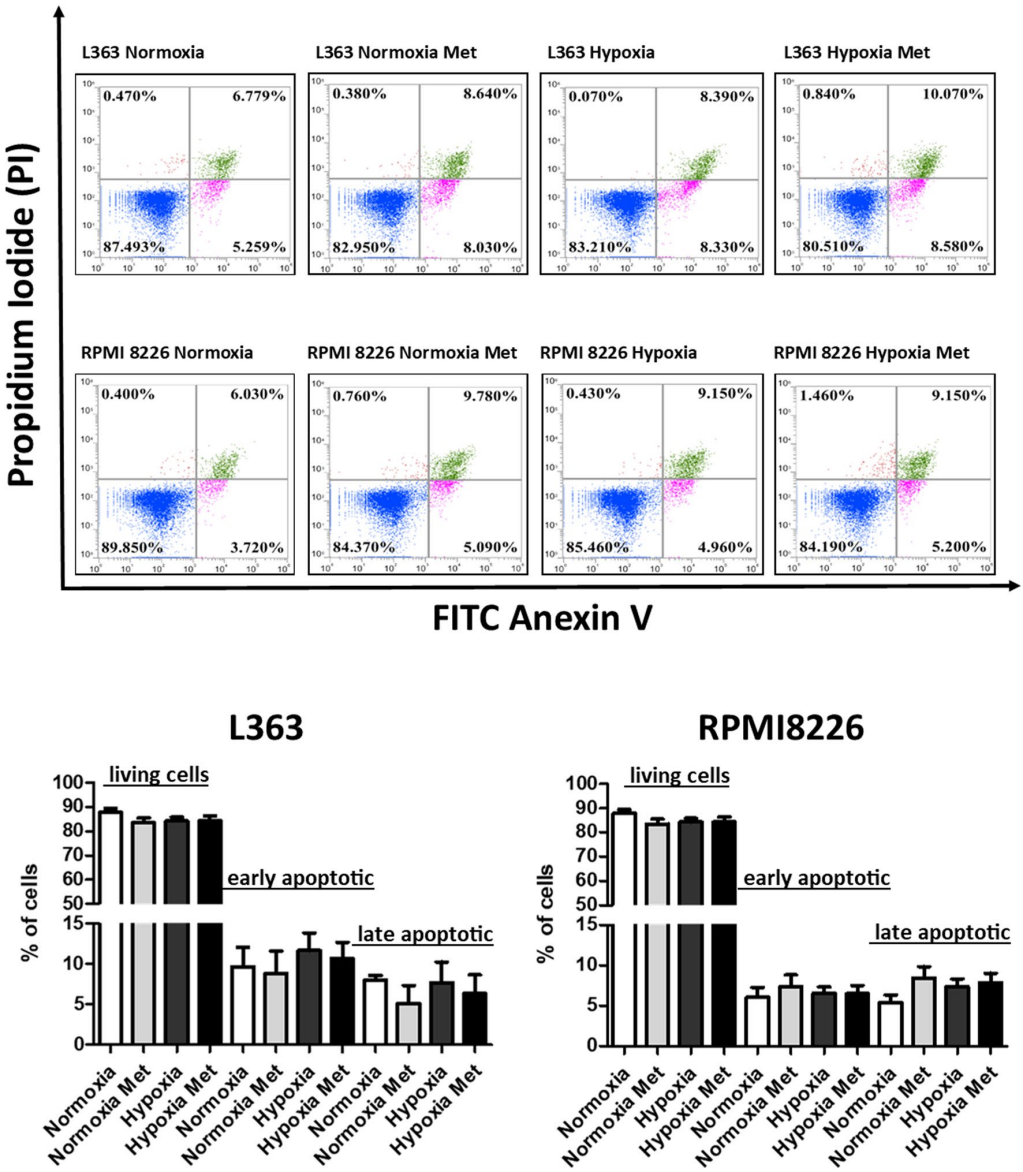
Effect of metformin on apoptosis induction in malignant plasma cells in normoxic and hypoxic conditions. L363 and RPMI 8226 cells were cultured for 48 h in hypoxic and/or normoxic conditions in the presence or absence of metformin (5 mM) and subsequently stained by Annexin V/PI followed by FACS analysis. The representative experiment of three independent ones is presented. The living cells were defined as Annexin V negative/PI negative, early apoptotic cells were defined as Annexin V positive/PI negative and late apoptotic cells were defined as Annexin V positive/PI positive. The % of early, late apoptotic cells and living cells in particular condition is given as the % of gated cells. The mean ± SEM of three independent experiments is shown. According to multiway ANOVA, there were no differences between % of living, early apoptotic and late apoptotic cells in different conditions

Next, to determine if the effect of metformin in normoxic and hypoxic conditions is a consequence of growth inhibition, apoptosis induction or both, we performed PI/Annexin V staining with subsequent FACS analysis. Using this approach living cells (Annexin V negative/PI negative,), were distinguished from early apoptotic cells (Annexin V positive/PI negative) and late apoptotic (Annexin V positive/PI positive) cells. As determined in Fig. 5, hypoxic stimulation, regardless the absence and presence of metformin, did not induce the apoptosis in analyzed cell lines. The lack of difference in the number of living cells, early apoptotic cells and late apoptotic cells between normoxic and hypoxic MM cells cultured in the presence and absence of metformin was confirmed using the multiway ANOVA.

## Discussion

**Scheme 1:**
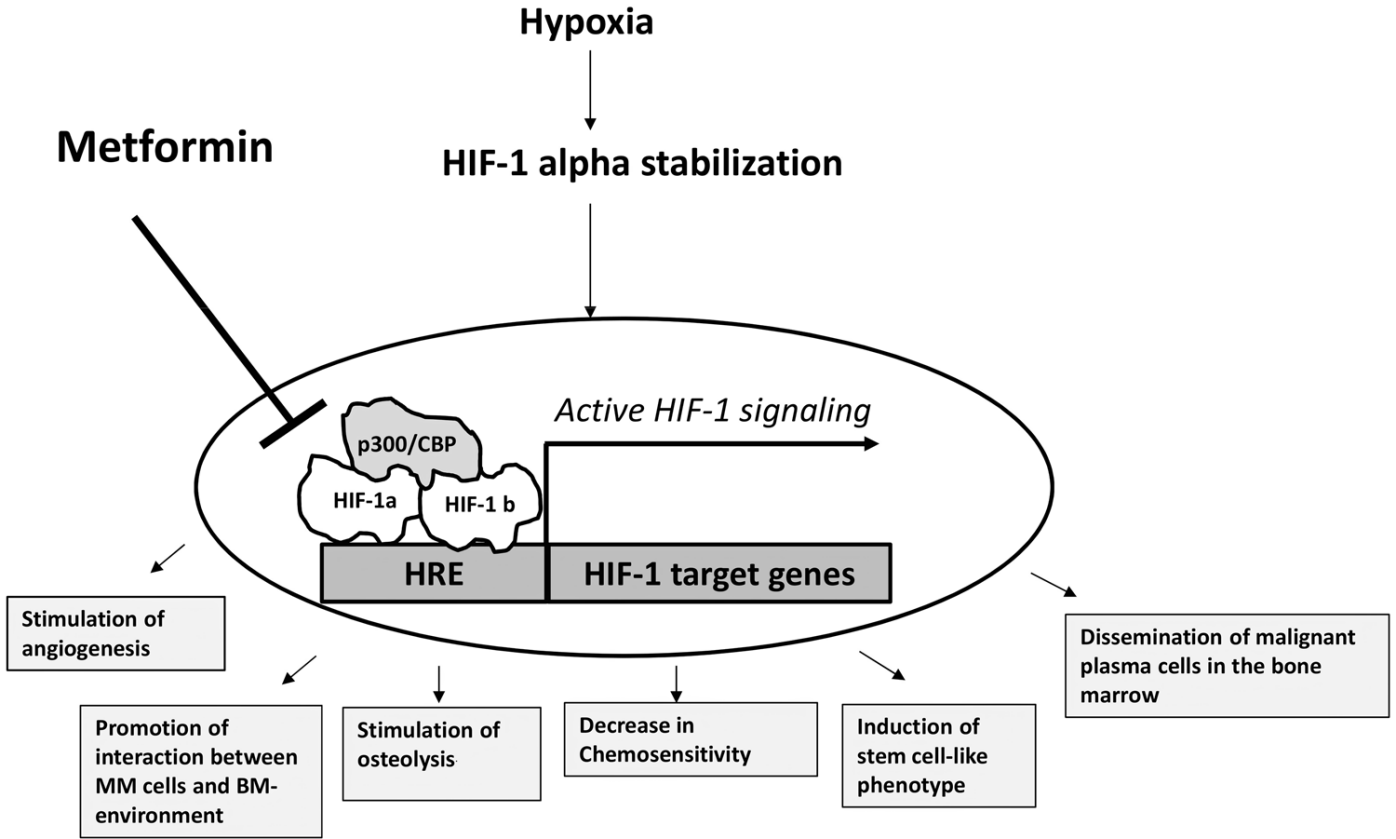
The significance of HIF-1 signaling for the progression of Multiple Myeloma

Importantly, the subsequent densitometry analysis revealed that induction of gene expression in hypoxic condition was significantly diminished by metformin (5 mM). Taking into consideration that hypoxia-triggered gene expression profile comprises many genes crucial for the progression of multiple myeloma [2–7, 17], one can hypothesize that addition of metformin to anti-MM therapy can be beneficial for MM patients. In Scheme 1 processes that could be potentially inhibited by metformin application are presented. As it was reported by Wheaton et al., metformin prevents hypoxic HIF-1 alpha accumulation via inhibition of mitochondrial respiratory chain complex I activity [18]. In consequence, the oxygen is shifted from mitochondria to cytosol allowing hydroxylation and subsequent degradation of HIF-1 alpha subunit [18]. Of note, the recently published study by Kurelac et al. confirmed that complex I deficiency results in HIF-1 alpha destabilization in hypoxic condition in parallel to an increase in oxygenation status in oxygen diminished environment [19]. Moreover, it has been shown by independent studies that elevation of alpha-ketoglutarate level, as a consequence of complex I impartment, increases the prolyl hydroxylase affinity for oxygen and as the result HIF-1α hydroxylation and degradation is promoted even at diminished environment [20]. In addition to mechanisms described above, Zhou et al. has shown that metformin can accelerate degradation of hypoxia-independent (Cobalt Chloride induced) stabilization of HIF-1 alpha subunit [11]. Nevertheless, as determined for L363 cell line in Fig. 1a (right panel) and 1B (lower panel), metformin may probably hinder, to some extent, also hypoxia-independent HIF-1 stabilization in multiple myeloma cells. Since physiological hypoxia appears mainly in short hypoxia-re-oxygenation cycles and in fact cycling hypoxia significantly contributes to cancer-induced angiogenesis [21], one could hypothesize/conclude that metformin, by inhibiting HIF-1 signaling in short hypoxia intervals, may significantly inhibit MM-induced angiogenesis and in consequence progression of the disease. In fact, the observed diminished effect of metformin on MM cells growth in hypoxic conditions can be explained by HIF-1 mediated growth inhibitory mechanism, which most probably does not exist in metformin treated hypoxic cells. Of note, it has been proved that HIF-1 induces growth arrest by stimulating the expression of p27 and p21 [22]. According to the study by Hackenbeck et al. the decrease in cell proliferation in hypoxia is fully dependent on HIF-1 signaling and not related to the oxygen-deprived environment [23]. Thus, it can be speculated that growth inhibition of malignant plasma cells in oxygen diminished environment most probably is the consequence of HIF-1 accumulation, whereas in hypoxic MM cells treated with metformin the growth inhibition can be related only to the action of metformin. Undoubtedly, one of the most important factors for effective application of metformin as an anti-cancer agent is the extent of its accumulation in particular tissues of patients treated by this drug. Although the concentration used in clinic is 1000 lower than the one used in in vitro studies, the epidemiological data clearly demonstrate that currently applied doses of metformin exert anti-cancer effect. Although more research is needed to address this issue, the fact that metformin concentration in mitochondrial matrix can reach even 1000-fold higher values than that observed in human serum [24, 25] indicates that this drug may inhibit HIF-1 and exhibit effective anti-MM activity, even in the currently clinically applied concentrations range. Taken together, these data demonstrate that metformin inhibits HIF-1 pathway in hypoxic malignant plasma and in consequence the signaling important for progression of the disease. Thus, the currently used therapeutic strategies may benefit from the addition of metformin, since several cancerogenesis related processes are known to be attenuated by inhibition of HIF-1 pathway.
